# Critically Ill Patients with Renal Hyperfiltration: Optimizing Antibiotic Dose

**DOI:** 10.1155/2023/6059079

**Published:** 2023-02-28

**Authors:** Jorge Rico-Fontalvo, José Correa-Guerrero, María Cristina Martínez-Ávila, Rodrigo Daza-Arnedo, Tomás Rodriguez-Yanez, Amilkar Almanza-Hurtado, José Cabrales, Carmen Julia Mendoza-Paternina, Alvaro Frías-Salazar, Julio Morales-Fernández

**Affiliations:** ^1^Colombian Association of Nephrology and Hypertension, Bogota, Colombia; ^2^Intensive Care Unit Universidad de Cartagena, Cartagena, Colombia; ^3^Epidemiology and Public Health, BIOTOXAM Group. Universidad de Cartagena, Cartagena, Colombia; ^4^Division of Nephrology, Stanford Healthcare/Stanford University School of Medicine, Stanford, CA, USA; ^5^Department of Medicine, Universidad del Sinu, Cartagena, Colombia; ^6^Intensive Care Unit Sanatorio Otamendi y Miroli Buenos Aires, Buenos Aires, Argentina

## Abstract

Renal hyperfiltration (RHF) is a prevalent phenomenon in critically ill patients characterized by augmented renal clearance (ARC) and increased of elimination of renally eliminated medications. Multiple risk factors had been described and potential mechanisms may contribute to the occurrence of this condition. RHF and ARC are associated with the risk of suboptimal exposure to antibiotics increasing the risk of treatment failure and unfavorable patient outcomes. The current review discusses the available evidence related to the RHF phenomenon, including definition, epidemiology, risk factors, pathophysiology, pharmacokinetic variability, and considerations for optimizing the dosage of antibiotics in critically ill patients.

## 1. Introduction

Renal hyperfiltration (RHF) is a recently described phenomenon in critically ill patients, which is defined as an augmented renal clearance (ARC) (creatinine clearance: CrCl) measured by 24-hour urine collection greater than 130 ml/min [1–3]. It is a clinical condition that has been gaining recognition in critical care; although it may have existed long before our current recognition, it was not until early 2010 that the research group led by Andrew Udy presented the concept as an existing medical phenomenon [4, 5]. It predominantly affects patients with severe neurological injuries, sepsis, trauma, and burns [1–6]^.^

RHF has been informed in approximately 30–65% of the universal population of patients hospitalized in the polyvalent intensive care unit (ICU), with a maximum incidence peak between the fourth and fifth day of admission and a tendency to stabilize nearby on the seventh day [6]. However, in certain specific populations, the incidence increases to 50–85% (i.e., patients with sepsis, subarachnoid hemorrhage, multiple traumas head trauma, or central nervous system -CNS- infections) [2, 5]. This high incidence emphasizes the importance of continuous evaluation of renal function to identify early the presence of RHF [1–6].

In a prospective study carried out in the ICU of the Otamendi Sanatorium, in Buenos Aires, between October 2011 and September 2012, an incidence of RHF of 28% was observed [4]. Age <48 years and the absence of comorbidities were the only variables that were independently associated with the development of RHF [4].

The development of systemic inflammatory response syndrome (SIRS) seems to be closely associated with RHF [6]. In septic patients, the mediators released with the inflammatory response can significantly increase cardiac output and downgrade systemic vascular resistance, which, added to fluid therapy and the use of vasoactive drugs, determine an increase in renal blood flow (RBF) and therefore of the glomerular filtration rate (GFR). These physiological changes predispose patients who are under antibiotic treatment to have subtherapeutic plasma concentrations of antibiotics; especially if these had renal elimination [3, 6, 7]. In the presence of RHF, the use of regular doses of antibiotics regimens could lead to the appearance of bacterial resistance and/or therapeutic failure, and a correlation with worse clinical results has been theorized [6, 7]. However, studies to evaluate the relationship between RHF and hard clinical outcomes such as mortality, ICU stay, the requirement for renal replacement therapy, vasopressors, and/or mechanical ventilation have failed [6, 7]. Therefore, would it be necessary to optimize the dosage of antibiotics in critically ill patients with RHF?

This narrative review aims to present the existing evidence related to the RHF, involving definition, epidemiology, determinants, pathophysiology, pharmacokinetic variability, its implications, and considerations for optimizing the prescription of antibiotics in ICU patients.

## 2. Definition

RHF is a newly termed phenomenon in critically ill patients, categorized by ARC and renal drug elimination, leading to suboptimal drug exposure, including antibiotics. This could be associated with an increase in the generation of bacterial resistance and potential therapeutic failure, thus worsening the prognosis of patients [1–6]^.^

Although there is no clear cut-off point for CrCl measured in 24-hour urine to define RHF, a level greater than 130 mL/min has been linked with subtherapeutic plasma concentrations of antibiotics, such as vancomycin and *β*-lactams, rising the risk of treatment failure and worse clinical results when these antibiotics are administered in standard doses [1–3]. A wide range of CrCl limits has been suggested to define RHF, ranging from 120 to >150 ml/min [2–4]. Specific patient populations, such as those with severe neurological injury, trauma, burns, and sepsis have been recognized as being at high risk for the development of RHF, with a mean CrCl ranging from 170 ml/min to over than 300 ml/min (Table 1) [1–6].

Another unresolved question is whether additional CrCl cut-off points are necessary to stage RHF equivalent to the commonly used classifications of renal dysfunction, i.e., mild, moderate, and severe RHF [2, 3].

Notwithstanding the various meanings observed and founded on the huge number of studies that used CrCl values >130 ml/min, as well as the clinical implications associated with this value, an integrated definition of RHF using CrCl >130 ml/min the adult population [1–6].

While conclusive screening tools are not yet proven, intensivists must be alert in identifying that RHF may be a causative issue disturbing the predictable outcomes of antibiotic treatment in patients [7].

### 2.1. Epidemiology

The prevalence of RHF has not been clearly defined. Although it has been reported in approximately 30–65%, which suggests that it is a frequent clinical phenomenon (Table 1) [1–6].

Campassi et al. carried out a prospective study between October 2011 and September 2012 in the ICU of the Otamendi Sanatorium, in which they showed an incidence of RHF of 28%, being the youngest age (<48 years) and the absence of comorbidities such as diabetes, the independent determining factors for the development of RHF [4].

Baptista et al. developed a retrospective cohort study with the objective of evaluating the risk factors for RHF and their prevalence in critically ill patients for one year [8]. The authors observed a prevalence of 24.9%, but in the subgroup of patients with normal serum creatinine, the diagnosis of HRH increased to 43%. Independent risk factors for RHF were young age, male gender, and trauma; particularly in those without evidence of renal dysfunction [8]. These findings were similar to the results in Campassi's work described previously [4].

The incidence could be as high as 50–85% in certain specific patient populations (i.e., sepsis, multiple traumas, head trauma, subarachnoid hemorrhage, central nervous system infections, and burns) [1–3].

The highest incidence of RHF in the development of life-threatening illnesses is during the first week of evolution in the ICU [7]. However, as it is considered a dynamic phenomenon and given the volatility of its length, uninterrupted monitoring of renal function and plasma levels of antibiotic concentrations would be justified, especially in patients with normal plasma creatinine values, to avoid therapeutic failures due to subtherapeutic drug levels [1–6].

**Figure 1:**
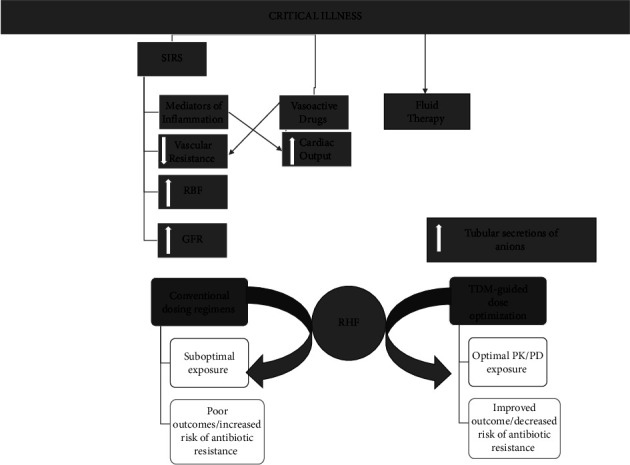
Causes and consequences of augmented renal clearance in critically ill patients. RBF: renal blood flow, GFR: glomerular filtration rate, TDM: therapeutic drug monitoring, PK/PD: pharmacokinetics/pharmacodynamics, SIRS: systemic inflammatory response syndrome. Modified from Sime FB [6].

**Figure 2:**
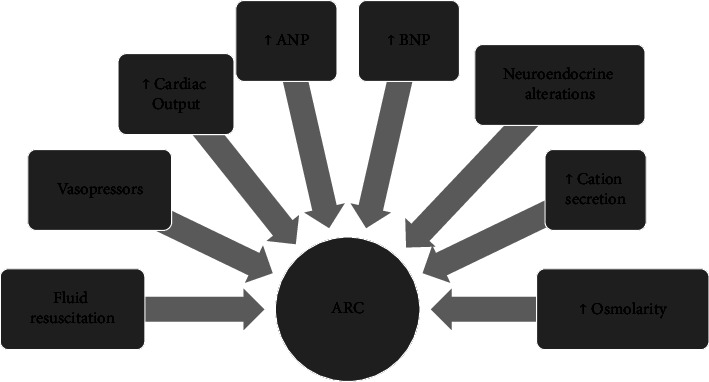
Potential contributing factors to ARC. ANP: atrial natriuretic peptide, ARC: augmented renal clearance, BNP: brain natriuretic peptide. Modified from Cook AM [1].

Although an absolute etiology has been hard to isolate, the development of SIRS appears to be closely associated with RHF [7] (Figure 1). Inflammatory mediators released by SIRS can substantively rise both cardiac output and capillary permeability and decrease systemic vascular resistance, producing an increase in renal blood flow (RBF) and glomerular filtration rate if renal function is intact (Figure 2).

In critically ill patients, resuscitative struggles such as management with fluids or vasopressors may also conduce to RHF, however, the relationship between vasoactive agents and RHF is still in analysis.

### 2.2. Risk Factors

Multiple studies compared critically ill patients with and without FHR revealing that younger age, male gender, diastolic blood pressure >65 mmHg, absence of comorbidities (i.e., chronic obstructive pulmonary disease, heart failure, diabetes mellitus, and hypertension), traumatic pathology as well as lower severity scores (SOFA, SAPS II, or APACHE II) were independent predictors associated with the development of RHF [1–6]. Likewise, RHF risk scoring systems have been developed with a sensitivity of 100% and specificity of 71.4%, a negative predictive value of 100%, and a positive predictive value of 75% [1–6].

Within the approach and study of RHF, the identification of high-risk subpopulations is essential to guide the application of predictive strategies that guide its diagnosis. An observational study carried out by the group led by Batista et al., with a 1-yearfollow-up, included 447 patients admitted to the ICU at a third-level hospital, and evaluated the characteristics with the capacity to predict the presence of RHF. Leading to the development of the ARCTIC (augmented renal clearance in trauma intensive Care) scoring system employed the patient with the following factors: serum creatinine, sex, and age to identify those with high ARC risk (ARCTIC score > 6)^8^. The ARCTIC scoring system produced a sensitivity of 84% and a specificity of 68% [8].

The usage of the ARC or ARCTIC predictive instruments permits the recognition of at-risk patients and guides clinicians to take suitable interventions (Table 2).

There are several patient groups that are related to an elevated prevalence of RHF, comprising patients with head trauma, major trauma, sepsis, ventilator-associated pneumonia, major surgery, CNS infection, burns, pregnant women, and hematological malignancy [3] (Table 1).

### 2.3. Pathophysiology

**Figure 3:**
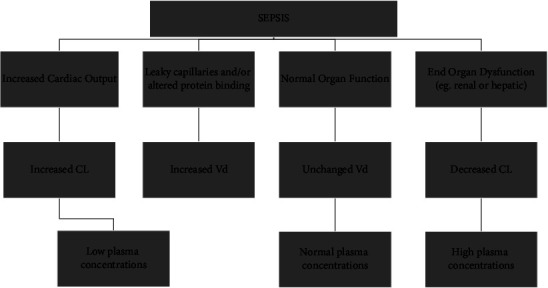
Diagrammatic view of pathophysiological variations that can happen during sepsis and their pharmacokinetic consequences. CL: clearance; Vd: volume of distribution. Modified from Roberts and Lipman [9].

A broad spectrum of pathophysiological changes can occur in the critically ill patient that has an impact on the pharmacokinetic and pharmacodynamic (PK/PD) properties of drugs [9]. In these patients, changes in the volume of distribution (Vd) and clearance occur that may affect the plasma concentrations of the antibiotics used. Consequently, it is essential to try to optimize antibiotic dosage regimens to maximize response rate and efficacy and minimize bacterial resistance leading to better patient outcomes [9] (Figure 3).

Actual knowledge of the pathophysiology of RHF is still scarce. It has been described to be correlated with renal tubular secretion of anions, renal tubular reabsorption, and increased glomerular filtration. The usage of exogenous markers suggests that RHF affects many mechanisms of the nephron, attributing changes in peripheral vascular permeability, increased RBF, and increased cardiac output secondary to SIRS triggered by an acute infection or even fever, as well as by other factors (acute brain injury, polytrauma, burns); resulting in a hyperdynamic state that can result in glomerular hyperfiltration manifesting as RHF [1, 2, 6, 9].

In addition, it is common for ICU patients to undergo fluid therapy and treatment with vasoactive and inotropic drugs, which could further contribute to the hyperdynamic state [6]. The exact mechanism linked to the pathophysiology of the RHF remains uncertain and is based on the results of experimental studies.

### 2.4. Antimicrobials in Critical Patients: Pharmacokinetics and Pharmacodynamics

Minimum inhibitory concentration (MIC) is an in vitro measure of antimicrobial effects on the pathogen thus it reflects the microbiological activity of antimicrobials [10]. PK/PD are tools that determine how much and how often the medication should be given. PK refers to the absorption, distribution, metabolism, and elimination of the medication, while PD defines the influence of the blood serum concentration and the reaction to the drug [10].

**Figure 4:**
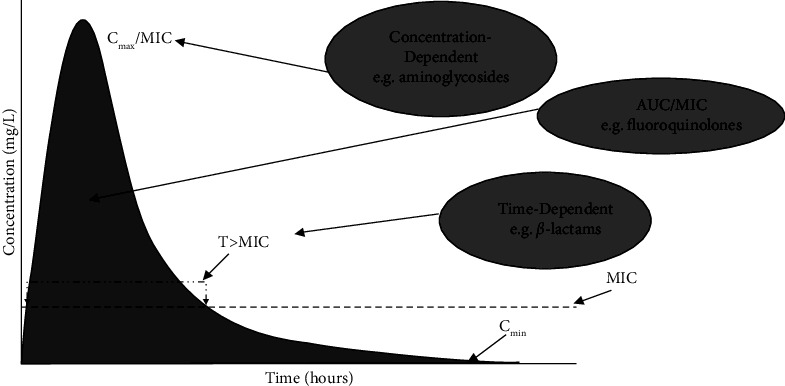
Correlation of the pharmacodynamics of an antimicrobial agent in terms of concentration vs time. *C*_max_/MIC: maximum concentration rate (*C*_max_) by MIC; *T* > MIC: time *T* that the antibiotic concentration remains above the minimum inhibitory concentration (MIC); MIC/AUC: ratio of the area under the curve (AUC) of concentration versus time above the MIC. Adapted from Freitas et al. [10].

According to the PD characteristics of an antibiotic, it can be cataloged as, concentration-dependent, time-dependent, and concentration/time-dependent as shown in Figure 4 and Table 3 [9, 10].

**Figure 5:**
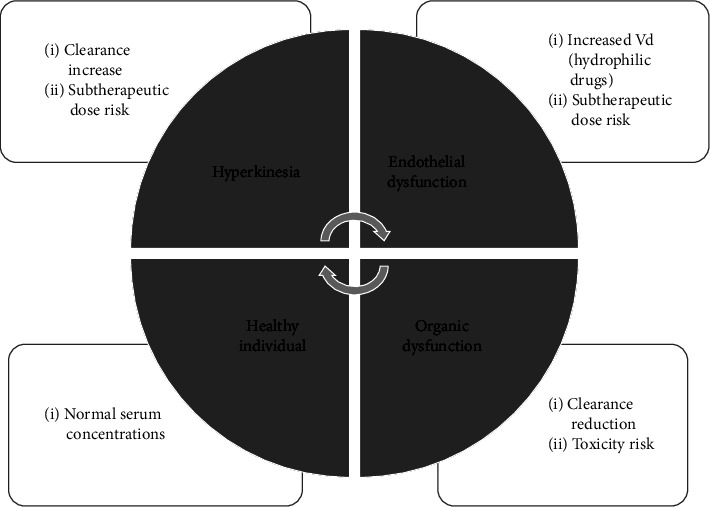
Clinical status of the patient and its influence on the pharmacokinetics of antimicrobials. Vd: volume of distribution. Modified from Freitas et al. [10].

Several mechanisms impact antimicrobic PK in critically ill patients (Figure 5) [9]. The distribution of.

Antimicrobial agents' distribution experiences deep changes during septic shock, there is an augmented production of endotoxins that can lead to the discharge of multiple inflammatory mediators that influence the vascular endothelium and conclude in low blood flow distribution due to increased capillary permeability, and endothelial injury. This promotes fluid extravasation from the intravascular to the interstitial space, rising the Vd of hydrophilic drugs, resulting in a decrease in their serum concentration, this can occur with glycopeptides, beta-lactam antibiotics, colistin, aminoglycosides, and linezolid (Table 4) [9]. The Vd can also be augmented in patients with hypoalbuminemia, under mechanical ventilation and being supported by extracorporeal circuits [5, 9, 10].

Among the considerations in relation to the dosage of antimicrobial agents, there is the location site of the infection, with a relevant variable such as the volume of distribution. For this reason, adequate doses of antibiotic agents must be guaranteed, especially in critically ill patients, where these concentrations could affect penetrance to the infection site [11]. By way of explanation, piperacillin has a plasma/pulmonary penetration ratio of 0.4-0.5 (free drug concentration), consequently, for pneumonia treatment, plasma concentration should be 200 to 250% of that required for bacteremia, with them to guarantee that antibiotic concentrations are equivalent at the site of infection [11].

### 2.5. Pharmacokinetic Changes in the RHF

The presence of RHF in critical patients can have a harmful influence on the achievement of therapeutic levels of many medications, so some investigation has been carried out to measure the impact on PK and clinical outcomes, almost all published references on this topic focus on antimicrobial therapy [1–3]. RHF influences the PK profile of antibiotics that are eliminated via the kidneys, especially those that have a direct connection between their renal metabolism and CrCl (vancomycin, aminoglycosides, or *β*-lactams); leading to a shorter half-life, an inferior *C*_max,_ and a lower AUC, having a direct implication in the PD effects affecting the efficacy as well as the increase in the appearance of bacterial resistance with drastic results in the evolution of the patient [1–3, 6].

### 2.6. Vancomycin Pharmacokinetics and RHF

Vancomycin is a hydrophilic, glycopeptide antibiotic that is eliminated unchanged renal pathways by 80–90%; it is used as the standard treatment of Gram-positive bacteria infections [12].

Numerous studies have been developed to determine the impact of RHF on the plasma concentration of vancomycin [2]. In a study carried out by Baptista et al., they reviewed the effect of RHF (urinary CrCl 130 ml/min) in 93 septic patients who received empirical or directed treatment with vancomycin in a continuous infusion, obtaining as a result that 37 patients with RHF got between 25 and 30% inferior levels of vancomycin with statistical significance (*P* < 0.05), and was powerfully linked with subtherapeutic serum concentrations in the first 72 hours of treatment [12].

In another study of the same group, they aimed to develop a nomogram for vancomycin dosage administered by continuous infusion during the first 24 hours of treatment, based on urinary CrCl measured over 8 hours [13]. In the first place, they retrospectively analyzed 79 patients, of whom 29 (36%) had RHF, and were treated with the hospital's standard vancomycin protocol; 8 (28%) out of the 29 patients with RHF reached the target level of plasma concentration of vancomycin 20–30 mg/L, compared with 64% (*n* = 32) of those without RHF (*P* = 0.092). Considering these results, the investigators elaborated a predictive equation for vancomycin clearance and a dosing nomogram based on urinary CrCl measured in 8-hour urine collections [13]. Applying this nomogram, it was possible to reach the therapeutic target level of vancomycin in 84% of patients. Patients included all those with RHF [13].

A prospective study run by Bilbao-Meseguer et al. confirmed the association between RHF and subtherapeutic levels of the plasma concentration of vancomycin in critical patients [14]. Thus, they demonstrated that patients with RHF had an inferior plasma concentration of vancomycin on the first days of treatment and no patient achieved the target level on the first day. This happens regardless of dose-increasing levels of vancomycin, which after 72 hours from the start of treatment were almost 50% higher than in patients without RHF [14]. Besides, at that time, mean plasma concentrations were beneath the lower limit of suggested levels. This implies that the majority of patients with RHF were treated improperly [14].

The study conducted by Spadaro et al. showed among patients with subtherapeutic levels of vancomycin, 50, 66, and 80% had RHF at the first monitoring (day 2), second (day 4) and third (day 6), respectively [14, 15]. Minkute obtained similar results and concluded that the risk of subtherapeutic levels of vancomycin is doubled in patients with FHR [16].

### 2.7. Impact of RHF on the Pharmacokinetics of *β*-Lactams

Since the early 1940s, *β*-lactams, owing to their broad spectrum of antibacterial activity, have been used as the keystone of antimicrobial treatments, for serious infections throughout the world [17]. Increases in resistance proportions have defied its well-known application in clinical practice. Therefore, continued infusion of *β*-lactam antibiotics has been recommended as one of the dosing strategies to improve PK/PD targets, and thus be able to improve the consequences of patients accepted to the ICU. Although the evidence on the efficacy of its administration in prolonged infusion is scarce, this measure is being adopted more and more in many ICUs worldwide.

*β*-Lactams are habitually used as empiric therapy in critical illnesses, have time-dependent antibacterial activity, are mainly eliminated via the kidneys, and are therefore affected by the presence of RHF, posing a significant risk to treatment success and patient outcomes [17]. Many studies informed treatment failures with *β*-lactams when using standard dosage regimens in patients with RHF, some of which are described as follows [2].

In a retrospective cohort conducted by Udy et al., they analyzed 52 trough concentrations of *β*-lactams, where piperacillin was the most prescribed (48%) [18]. Most patients were on mechanical ventilation on the day of the study (85%), and 25% received vasopressor support. Just 30 patients (58%) had a trough drug concentration equal to or greater than the MIC, a number that decreased to 16 (31%) when targeting four times the MIC [18]. The presence of RHF was related to trough concentrations lower than the MIC or less than four times in 82 and 72% of cases, respectively. Multivariate analysis confirmed that CrCl remains a significant predictor of the probability of obtaining subtherapeutic levels of *β*-lactams [18].

Subsequently, in 2015, the same author carried out another study, in this case, a single-center observational study, with the objective of exploring the impact of RHF and the dissimilar susceptibilities of bacteria in achieving the PK/PD objective of piperacillin (time above MIC (fT > MIC)) in critically ill patients with sepsis receiving intermittent dosing [19]. The results indicate that an empirical intermittent infusion of 4.5 g piperacillin/tazobactam is improbable to reach ideal piperacillin exposure in an important proportion of patients, especially when they target less susceptible pathogens [19].

Casu et al. investigated whether variation in CrCl correlates with changes in *β*-lactam concentrations or pharmacokinetics in septic patients [20]. Obtained data from 56 adult patients admitted to the ICU in whom MTD of broad-spectrum antibiotics (ceftazidime, cefepime, piperacillin, or meropenem) were performed, showed that the PK of *β*-lactams is significantly altered by CrCl (>125–130 ml/min) leading to an insufficient therapeutic level in more than 50% of patients [20]. However, the main finding was that dose adjustment is not reliable for changes in kidney function alone. Therefore, the MTD of *β*-lactams is still necessary to optimize antimicrobial dosing strategies [20].

Carlier et al. in a prospective, observational study, evaluated the influence of RHF on the achievement of the PK/PD goal in critically ill patients getting piperacillin/tazobactam or meropenem, given as a prolonged infusion [21]. This study showed that 48% of patients did not reach the desired PK/PD goal, of which almost 80% had RHF [21]. Subsequently, these patients may be at threat of treatment failure if there's not a dose increase [21].

RHF as a predictor of subtherapeutic levels in trauma patients receiving piperacillin/tazobactam was Akers et al. main purpose study [22]. Patients were categorized into two risk groups (low: 0–6 points, high: >6 points) founded on the RHF score (Table 2) proposed by Hobbs et al. [3]. The ARC score was 100% sensitive and 71.4% specific for detecting an increase in CrCl [22]. In addition, authors detected a greater Vd, decreased in AUC, and fT > MIC < 50% at a MIC of 16 *μ*g/ml^22^. The area under the receiver operating characteristic curve was 0.86 for each, reflecting a high degree of diagnostic accuracy for the ARC score [22]. Serum creatinine less than 0.6 mg/dL had comparable specificity (71.4%) but was less sensitive (66.7%) and accurate (area under the ROC curve 0.69) in detecting higher clearance rates [22].

*β*-lactams are agents that act over time above the minimum inhibitory concentration, therefore in the RHF scenario, they may require higher doses to perform their function adequately. On the other hand, adverse clinical results have been identified in patients with subtherapeutic concentrations of these agents. For this reason, the individualization of drug administration regimens based on closed monitoring of their serum levels is proposed, accompanied by their use in a continuous infusion, maximizing the possibility of achieving therapeutic goals, and eradicating the infection.

### 2.8. Other Antibiotics

Aminoglycosides are eliminated primarily via the kidneys with expectable efficacy founded on serum concentrations. In a 204 patients retrospective analysis performed by Goboova et al., patients received gentamicin, of which 29 patients (14%) had ARC [23]. Analysis of gentamicin Cmax found that subtherapeutic concentrations occurred in 93% of patients who had RHF requiring a dose increase to reach maximum target levels [23]. The results of this study highlight the role of drug monitoring in the context of RHF.

Another affected family of antibiotics affected by RHF is fluoroquinolones, therefore MTD could be valuable when used in these patients with RHF, as superior doses may be required [2]. As an instance, supported by Monte Carlo simulation, in patients with *S. aureus*, S. *pneumoniae,* and *P. aeruginosa* infections and CrCl >130 mL/min, a superior levofloxacin dose of 1 gram every 24 hours have been proposed (conventional dosing 0.5–0.75 g every 24 h) [2].

### 2.9. Dosage Considerations

The implications of RHF for antibacterial dosing strategies in the critically ill will depend significantly on the specific “death characteristic' of any given agent [5]. In general terms, this determines that PK/PD is the factor most closely associated with effective bacterial eradication [5].

### 2.10. Application in Clinical Practice

To determine antimicrobial therapy outcome there are three factors to be considered host (patient), bacteria, and antibiotic [24]. These aspects determine the successful dose needed to eradicate the infection without harming the patient [24]. As mentioned before critically ill patients have distorted pharmacokinetic parameters and are usually infested by more resistant microorganisms [6], hence a personalized method to achieve correct dosing for each patient is required [25]. Precision medicine offers it and incorporates precision dosing through therapeutic drug monitoring (TDM) [26].

The current evidence does not support the use of algorithms or precise adjustment models for the dosage of antimicrobials in critically ill patients with RHF, it is necessary to develop complementary studies from the pharmacokinetics and the clinic for these agents and estimate their real impact on adverse results. For this reason, derived from the currently available data, there are few guidelines that can be delivered to clinicians for the dosage and titration of antibiotics in critical scenarios. One proposal would be the use of follow-up by TDM, still under construction.

An approach based on personalized medicine, seeking to identify patients with RHF, forces us to start from the identification of the risk factors associated with the development of this condition, identifying groups of patients susceptible to complementary studies [3, 8]. In this high-risk subpopulation, it is proposed to carry out serial creatinine clearance medications, recommending measurements for 8 to 24 hours [2]. Once confirmed ARC, considerations of PD/PK should be made for all renally cleared medications [27]. Highlighting that aspects such as protein binding and Vd determine the impact of renal function on its elimination and therefore on its serum levels [27]. In renally cleared medications monitor plasma levels, if possible [28].

However, we must point out the limitations regarding the widespread availability of TDM for routine use in ICUs. With limited evidence, to guide dosage modification in the ARC population, an alternative, evaluating risk-benefit and the patient's condition, is to use higher doses of antimicrobial agents proportional to the estimated increased renal losses; this could be done using mathematical models or monograms [25, 28]. Always with close clinical monitoring [25, 28]. Assess the possibility of restricting the gaps of administration and using extended or continuous infusions in time-dependent antibiotics [24, 27]. Contemplate switch to an alternative drug agent with other routes of elimination, not largely renally eliminated [24].  Table 5 shows recommended initial dosing of studied medications in patients with ARC [28].

In order to improve the TDM of antibiotics in critically ill patients, future perspectives point to the development of rapid bioanalytical techniques that can be used simply, with cheaper equipment or with real-time monitoring of drug concentrations through means of biosensors at the patient's bedside [27].

## 3. Conclusion

Antibiotic treatment in critically ill patients continues to be a major challenge for ICU doctors worldwide, hence the importance of effective therapy consisting of control of the infectious focus, and early and suitable antibiotic treatment, these being the paramount interventions that the physician can apply.

RHF is a new phenomenon, without a unanimously recognized etiology and with diverse incidence proportions that are pronounced more frequently in specific groups of patients hospitalized in intensive care. With respect to pathophysiology, it induces important changes in PK/PD, thus hindering the therapeutic objective. In the case of sepsis, an insufficient antibiotic dose aggravates the prognosis and increases the generation of resistance. Therefore, the optimization of the dosage in patients with RHF should be guided and adjusted by serum therapeutic monitoring, based on the PK/PD properties, this being of main importance in the treatment of ICU patients.

## Figures and Tables

**Table 1:** Populations at risk of RHF. Adapted from Cook and Hatton-Kolpek [1].

Population	Prevalence (%)	Average CrCl value (ml/min)
Burned (burned surface ranged from 33 ± 21.3 percent of total body surface)	65	172.1
Sepsis	39.5–56	154–210
Subarachnoid hemorrhage	100	326
Febrile neutropenia	16.4	157.4
Trauma	85.7	166
Traumatic brain injury	85	150 (while not receiving CPP treatment)

CrCl: creatinine clearance; CPP: cerebral perfusion pressure.

**Table 2:** The ARC risk scoring systems. Modified from Hobbs et al. [3] and Baptista et al. [8].

	ARC scoring system	ARCTIC scoring system
Criteria	Age ≤ 50 years = 6 points	SCr < 62 *μ*mol/L = 3 points
	Trauma on admission = 3 points	Male sex = 2 points
	SOFA ≤ 4 = 1 point	Age < 56 years = 4 points
		Age: 56–75 years = 3 points

Interpretation	0–6 points: low risk	<6 points: low ARC risk
	7–10 points: high-risk	>6 points: high ARC risk

Sensitivity	100%	84%

Specificity	71%	68%

**Table 3:** Efficacy of certain antibiotics based on pharmacodynamic properties. Modified from Roberts and Lipman [9].

Antibiotics	*β*-lactams	Aminoglycosides	Fluoroquinolones
		Metronidazole	
	Carbapenem	Telithromycin	Aminoglycosides
	Linezolid	Daptomycin	Azithromycin
	Erythromycin	Quinupristin/dalfopristin	Tetracyclines
	Clarithromycin		Glycopeptides
	Lincosamides		Tigecycline

PD kill characteristics	Time-dependent	Concentration-dependent	Concentration-dependent with time-dependence

Optimal PD parameter	fT > MIC	*C*_max_/MIC	AUC 0–24/MIC

MIC: minimum inhibitory concentration; AUC: area under the curve, PD: pharmacodynamics, *C*_max_: maximum concentration, fT > MIC: free drug concentration above time.

**Table 4:** Behavior of the pharmacokinetic characteristics of hydrophilic and lipophilic antibiotics in patients in the general ward and in the critical care unit.

	Hydrophilic antibiotics	Lipophilic antibiotics
General ward PK	(1) Low intracellular penetration	(1) High Vd
	(2) Predominant renal CL	(2) Predominant hepatic CL: needs liver metabolism to render them water soluble
	(3) Low Vd	(3) Good intracellular penetration

Altered PK in ICU	(1) ↑ Vd	(1) Vd largely unchanged
	(2) CL ↑ or ↓ dependent on renal function	(2) CL ↑ or ↓ dependent on liver function

Examples	Fluoroquinolones: levofloxacin, ciprofloxacin	Fluoroquinolones: moxifloxacin, gatifloxacin, sparfloxacin, tigecycline, lincosamides, and macrolides
	*β*-lactams	
	Colistin	
	Aminoglycosides	
	Glycopeptides	
	Linezolid: nonhepatic nor renal elimination	

PK: pharmacokinetics Vd: volume of distribution. CL: clearance. Modified from Roberts and Lipman [9].

**Table 5:** Antibiotic dosage in patients with RHF. Modified from Tomasa Irrigable [28].

Drug	Usual doses (normal GFR) (Mesa 2017)	Suggested dose for HRF	Special cases
Levofloxacin	500 mg IV every 24 hours	750 mg IV every 24 hours	For *S*. *pneumoniae* infections, *P. aeruginosa* and *S. aureus* 1,000 mg/24 h iv
Meropenem	1 g IV every 8 hours	2 g IV C/8 h2	Doses of up to 8 g per day may be required [2]
Cefepime	1-2 g IV every 8–12 hours	2 g IV every 8 h prolonged infusion 3 h^3^	
Piperacillin/Tazobactam	2–4 g IV every 6–8 hours maximum dose 4 g IV every 4 h	4.5 g/4–6 hrs prolonged infusion of 4 h^2^	36 g/day may be needed to achieve the therapeutic goal
Vancomycin	15–20 mg/kg/every 8–12 noon IV Maximum 4 g/day	45 mg/kg/day spread over 3 doses or in continuous infusion	According to the nomogram and RHF stratification, if mild RHF (CrCl 130–150 ml/min) give the highest recommended dose habitually. If moderate RHF (CrCl 150–200 ml/min) give between 3 and 4 g/day. If high ARC (CrCl 200–250 ml/min) give between 4 and 4.5 g/day. If very high ARC (CrCl 250–300 ml/min) give between 4.5 and 5.5 g/day If extreme ARC (CrCl > 300 ml/min) give 6 g/day

## Data Availability

The data that support the findings of this study are available from the corresponding author upon reasonable request.
